# Effects and mechanisms of mindfulness training and physical exercise on cognition, emotional wellbeing, and brain outcomes in chronic stroke patients: Study protocol of the MindFit project randomized controlled trial

**DOI:** 10.3389/fnagi.2022.936077

**Published:** 2022-09-29

**Authors:** Adrià Bermudo-Gallaguet, Mar Ariza, Rosalia Dacosta-Aguayo, Daniela Agudelo, Neus Camins-Vila, Maria Boldó, Òscar Carrera, Sandra Vidal, Blai Ferrer-Uris, Albert Busquets, Marc Via, Guillem Pera, Cynthia Cáceres, Meritxell Gomis, Alberto García-Molina, José María Tormos, Ana Arrabé, Gustavo Diez, Maria José Durà Mata, Pere Torán-Monserrat, Juan José Soriano-Raya, Sira Domènech, Alexandre Perera-Lluna, Kirk I. Erickson, Maria Mataró

**Affiliations:** ^1^Department of Clinical Psychology and Psychobiology, University of Barcelona, Barcelona, Spain; ^2^Institut de Neurociències, University of Barcelona, Barcelona, Spain; ^3^Institut de Recerca Sant Joan de Déu, Esplugues de Llobregat, Spain; ^4^Department of Medicine, University of Barcelona, Barcelona, Spain; ^5^Unitat de Suport a la Recerca Metropolitana Nord, Fundació Institut Universitari per a la Recerca a l’Atenció Primària de Salut Jordi Gol i Gurina, Mataró, Spain; ^6^Institut Nacional d’Educació Física de Catalunya, University of Barcelona, Barcelona, Spain; ^7^Department of Physical Medicine and Rehabilitation, Hospital Universitari Germans Trias i Pujol, Badalona, Spain; ^8^Germans Trias i Pujol Research Institute, Hospital Universitari Germans Trias i Pujol, Badalona, Spain; ^9^Department of Neurosciences, Hospital Universitari Germans Trias i Pujol, Badalona, Spain; ^10^Institut Guttmann, Institut Universitari de Neurorehabilitació, Universitat Autònoma de Barcelona, Badalona, Spain; ^11^Nirakara Lab, Mindfulness and Cognitive Science Extraordinary Chair, Universidad Complutense de Madrid, Madrid, Spain; ^12^Institut de Diagnòstic per la Imatge, Hospital Universitari Germans Trias i Pujol, Badalona, Spain; ^13^B2SLab, Departament d’Enginyeria de Sistemes, Automàtica i Informàtica Industrial, Universitat Politècnica de Catalunya, Barcelona, Spain; ^14^Department of Psychology, University of Pittsburgh, Pittsburgh, PA, United States; ^15^PROFITH “PROmoting FITness and Health Through Physical Activity” Research Group, Sport and Health University Research Institute (iMUDS), Department of Physical and Sports Education, Faculty of Sport Sciences, University of Granada, Granada, Spain

**Keywords:** stroke, mindfulness, physical exercise, computerized cognitive training, neuroimaging, biomarkers

## Abstract

**Background:**

Post-stroke cognitive and emotional complications are frequent in the chronic stages of stroke and have important implications for the functionality and quality of life of those affected and their caregivers. Strategies such as mindfulness meditation, physical exercise (PE), or computerized cognitive training (CCT) may benefit stroke patients by impacting neuroplasticity and brain health.

**Materials and methods:**

One hundred and forty-one chronic stroke patients are randomly allocated to receive mindfulness-based stress reduction + CCT (*n* = 47), multicomponent PE program + CCT (*n* = 47), or CCT alone (*n* = 47). Interventions consist of 12-week home-based programs five days per week. Before and after the interventions, we collect data from cognitive, psychological, and physical tests, blood and stool samples, and structural and functional brain scans.

**Results:**

The effects of the interventions on cognitive and emotional outcomes will be described in intention-to-treat and per-protocol analyses. We will also explore potential mediators and moderators, such as genetic, molecular, brain, demographic, and clinical factors in our per-protocol sample.

**Discussion:**

The MindFit Project is a randomized clinical trial that aims to assess the impact of mindfulness and PE combined with CCT on chronic stroke patients’ cognitive and emotional wellbeing. Furthermore, our design takes a multimodal biopsychosocial approach that will generate new knowledge at multiple levels of evidence, from molecular bases to behavioral changes.

**Clinical trial registration:**

www.ClinicalTrials.gov, identifier NCT04759950.

## Introduction

Stroke is a leading cause of disability worldwide (Feigin et al., 2017). Advances in acute treatment have increased the number of people surviving a stroke which, in turn, has led to a growth in the number of patients with long-term consequences. It is estimated that up to 50% of stroke survivors experience some degree of disability at chronic stages of the disease, which jeopardizes functionality and quality of life (Donkor, 2018). This burden affects the daily life of survivors and their families and implies high health costs for governments (Rigby et al., 2009; Donkor, 2018).

Post-stroke cognitive and emotional complications are frequent consequences in the chronic stages of the disease. The prevalence of cognitive deficits one year after stroke ranges from 30 to 60%, depending on the study (Sexton et al., 2019). Furthermore, stroke significantly increases the risk of dementia in the long term (Pendlebury and Rothwell, 2009). Regarding emotional wellbeing, post-stroke depression and anxiety are common, being present in approximately one-third of the patients at any point in time (Ayerbe et al., 2013). Both cognitive and emotional disturbances are related to poor rehabilitation outcomes, morbidity, and mortality (Ayerbe et al., 2013; Obaid et al., 2020).

Consequently, it is fundamental to explore and test new health-behavior strategies to improve cognition and emotional wellbeing and prevent progressive cognitive decline and neurodegeneration after stroke. These strategies must be high-impact, low-cost, and easily accessible and implemented. Mindfulness, physical exercise (PE), and computerized cognitive training (CCT) have promising therapeutic potential in this scenario. These may benefit stroke patients by impacting neuroplasticity and brain health (Su and Xu, 2020).

Mindfulness is defined as “paying attention in a particular way: on purpose, in the present moment, and non-judgmentally” (Kabat-Zinn, 2003). Standardized interventions based on this concept [e.g., mindfulness-based stress reduction (MBSR)] have grown substantially in recent decades and have been successfully applied in healthy and clinical human populations, with benefits reported in a broad range of variables: mental health, cognition, pain, fatigue, physical health, and quality of life (Goldberg et al., 2022). In stroke, previous systematic reviews emphasize the potential utility of mindfulness in treating emotional disturbances (Lawrence et al., 2013), fatigue (Ulrichsen et al., 2016), and sensorimotor functions (Zou et al., 2018), while its benefits to cognition are still understudied.

The effects of mindfulness-based interventions may be explained by multiple biological pathways involving the cardiovascular, metabolic, inflammatory, and neuroendocrine systems (Reive, 2019). In healthy and clinical populations, the most frequently reported findings (not always in a consistent way) include reductions in circulating C-reactive protein (CRP), interleukin-6 (IL-6), and cortisol, and increases in brain-derived neurotrophic factor (BDNF) and insulin-like growth factor I (IGF-I) (Sanada et al., 2017; Reive, 2019). Meanwhile, neuroimaging studies have described structural and functional brain correlates of mindfulness in areas and networks related to attentional control, emotional regulation, and self-awareness (e.g., prefrontal and anterior cingulate cortex, amygdala, insula) (Tang et al., 2015). However, neither blood biomarkers nor brain data correlates of mindfulness have been addressed yet in stroke.

Physical exercise is considered a “planned, structured, and repetitive subtype of physical activity that aims to improve physical fitness” (Caspersen et al., 1985). Multiple systematic reviews and meta-analyses have shown the benefits of PE on cognition and emotional wellbeing across different age groups and health states (Rebar et al., 2015; Stillman et al., 2020). In stroke patients, contrarily to the effects of mindfulness, the gains of PE on cognition and emotional wellbeing have been more thoroughly studied. Some meta-analyses of randomized controlled trials have shown small-to-moderate effects of PE on cognition (Oberlin et al., 2017), depressive symptoms (Eng and Reime, 2014), and post-stroke health-related quality of life (Ali et al., 2021).

There might be a variety of processes behind the benefits of PE, including cellular and molecular, brain structure and function, and psychosocial mechanisms (Stillman et al., 2020). Biological mechanisms underlying cognitive and emotional benefits of PE include increased production of growth factors (e.g., BDNF, IGF-1), and decreased synthesis of pro-inflammatory molecules (e.g., CRP, IL-6), and accumulation of markers of neurodegeneration (Kandola et al., 2019; Stillman et al., 2020; Erickson et al., 2022). At a brain level, the positive influence of PE on the hippocampus and prefrontal gray matter volume (Erickson et al., 2014), global white matter volume (Sexton et al., 2016), and increased cross-network specificity across various functional networks (Stillman et al., 2019) have been described. Nevertheless, it should be noted that most of this evidence comes from studies with healthy older adults. In stroke, knowledge is still scarce, with preliminary findings in animal models and clinical studies focusing mainly on motor recovery (Xing and Bai, 2020).

Computerized cognitive training consists of repeated practice of standardized cognitive exercises that can be performed through the computer or mobile technology (Gates and Valenzuela, 2010). CCT has rapidly evolved in the last decade because of its potential to systematically apply personalized cognitive rehabilitation and its straightforward implementation in at-home use (Solana et al., 2015). In stroke care, multiple systematic reviews and meta-analyses have addressed the potential benefits of CCT in cognitive functioning (Mingming et al., 2022). However, although promising, results remain inconclusive due to study heterogeneity and methodological limitations. Some authors have further analyzed the additive gains of combining CCT with PE in stroke patients, with preliminary positive results (Amorós-Aguilar et al., 2021).

From a molecular and brain perspective, applying CCT following a stroke is based on the idea that repetitive cognitive tasks might promote neuroplasticity mechanisms and restore impaired cognitive function (Sigmundsdottir et al., 2016). Based on animal models using enriched environments, some authors hypothesize that cognitive stimulation promotes the release of growth factors (e.g., BDNF and nerve growth factor) which, in turn, enhances cell survival and proliferation in human brains (Valenzuela et al., 2007). Few studies have shown structural and functional brain changes after applying CCT to stroke (van de Ven et al., 2016), while others have fallen to prove any changes in neuroimaging data (Nyberg et al., 2018).

To conclude, cumulative evidence indicates that mindfulness, PE, and CCT could be effective strategies against post-stroke cognitive impairment and emotional disturbances. Nonetheless, it is necessary to conduct new randomized controlled trials to confirm and deepen these benefits and elucidate the biological mechanisms (i.e., metabolic, immune, cardiovascular, neuroendocrine) by which they occur. As a step forward, the inclusion of gut microbiome data, which seems to be affected in stroke patients (Lee et al., 2021) and might be modified by lifestyle behaviors (Manor et al., 2020), will offer new and valuable insights into stroke pathophysiology and rehabilitation.

## Aims of the study

The primary objective of the MindFit Project is to examine the effects of MBSR and PE on cognitive and emotional outcomes in chronic stroke patients by comparing three groups of participants who receive: MBSR + CCT, PE + CCT, or CCT alone. The primary hypotheses sustaining our main objective are:

(1) All three groups will experience gains at 3 months compared to baseline in cognitive functions and emotional status.
(2) Given the study design (i.e., all three groups receive the same CCT treatment, with the only difference being the addition of either MBSR or PE), these improvements in cognition and emotion will be greater in the combined treatment groups than in the only CCT group (either to a greater extent in the same outcomes or different cognitive and emotional measures).
(3) The add-on effects of MBSR and PE will differ from each other (i.e., they will have a specific profile of cognitive and emotional gains). Although there is a lack of studies comparing mindfulness and PE, we expect greater emotional gains after MBSR than PE, while PE will produce more cognitive benefits than MBSR.

The secondary objectives of the MindFit Project are:

(a) To study intervention-related changes in the molecular-cellular level (e.g., cardiometabolic, inflammatory, neurotrophic factors, gut microbiome species), the brain level [i.e., structural and functional magnetic resonance imaging (MRI)], and the behavioral level (e.g., physical activity and fitness status, mindful thinking, sleep quality, fatigue, quality of life).
(b) To examine whether changes in the molecular-cellular, brain and behavioral levels mediate cognitive and emotional intervention-related changes.
(c) To determine the genetic [i.e., apolipoprotein E (APOE) genotype], clinical (e.g., comorbidities, level of brain atrophy), sociodemographic (e.g., sex, age, educational level), and individual factors (e.g., cognitive reserve, general intelligence, personality), that moderate cognitive and emotional intervention-related changes.

**FIGURE 1:**
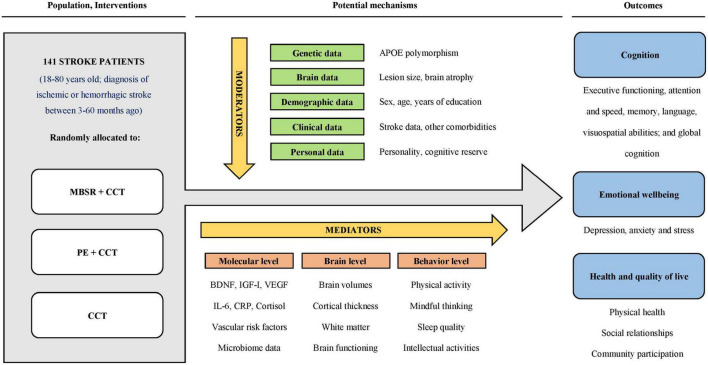
MindFit project graphical objectives. APOE, apolipoprotein E; BDNF, brain-derived neurotrophic factor; CCT, computerized cognitive training; CRP, C-reactive protein; IGF-I, insulin-like growth factor I; IL-6, interleukin-6; MBSR, mindfulness-based stress reduction; PE, physical exercise; VEGF, vascular endothelial growth factor.

A graphical representation of our objectives is presented in Figure 1.

## Materials and methods

### Study oversight and schedule

**FIGURE 2:**
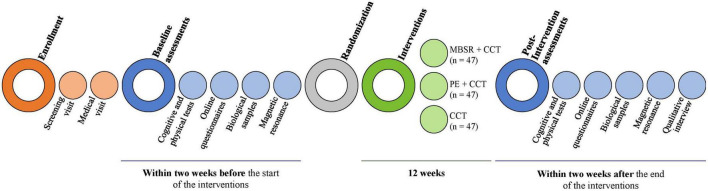
MindFit project study design. CCT, computer-based cognitive training; MBSR, mindfulness-based stress reduction; PE, physical exercise.

The MindFit Project is a prospective, parallel, three-arm, single-blinded randomized controlled trial. The trial consists of three groups in total, of which two are intervention groups, and one is the active control group. One hundred forty-one participants at three to 60 months after stroke are randomly allocated to one of three arms (1:1:1 allocation ratio). The first intervention group receives an MBSR program combined with CCT; the second intervention group receives a multicomponent PE program combined with CCT; and the third group, as an active control group, receives only CCT. The interventions last 12 weeks, with assessments at baseline and within two weeks after completion of the trial (see Figure 2). This study is led by the Faculty of Psychology of the University of Barcelona in collaboration with Hospital Universitari Germans Trias i Pujol and Institute Guttmann. The responsible ethics committees approved it following the Declaration of Helsinki. This protocol follows the SPIRIT recommendations (Chan et al., 2013).

### Deviation from the initial protocol

The MindFit Project was initially planned to be entirely onsite. However, all interventions and most assessments (i.e., all of them except for biological sample collection and brain scans) had to be converted to online versions because of the COVID-19 pandemic. The biological sample collection laboratory and the MRI center remained open with extra protective measures during the pandemic.

We followed international recommendations to adapt assessments and interventions to the virtual context. Furthermore, we ran several pilot groups to practice and improve our protocols according to participants’ feedback. Finally, the project started in November 2020, and data collection was completed in December 2021 (see NCT04759950 in www.ClinicalTrials.gov for the entire study version history; first posted on February 18, 2021).

### Participants

Inclusion and exclusion criteria are detailed in Table 1. Based on stroke epidemiology (Feigin et al., 2017), we anticipated approximately 60% of the sample to be men and 80% of cases to be ischemic strokes.

**TABLE 1:** Eligibility criteria.

Inclusion criteria	Exclusion criteria
• Women and men aged 18–80 years old • Diagnosis of ischemic or hemorrhagic stroke • Stroke diagnosis between 3 and 60 months ago • To have consent from a physician to engage in an exercise intervention • Fluency in Catalan or Spanish (i.e., able to understand and speak) • Accept to participate in the study and sign the informed consent according to the Declaration of Helsinki	• Cognitive impairment defined as a score in Mini-Mental State Examination ≤23 (Blesa et al., 2001) • Severe aphasia defined as a score ≥2 in item 9 of the National Institute Health Stroke Scale (Montaner and Alvarez-Sabín, 2006) • Severe sensory problems • Diagnoses of transient ischemic attack without evidence of brain lesion • Other neurological conditions apart from stroke (e.g., traumatic brain injury, brain tumors) • Severe pre-stroke psychiatric disorders (e.g., bipolar disorder, schizophrenia) • History of alcohol or other substance abuse

After the recruitment phase, we achieved our desired sample size. One hundred forty-one stroke patients from Catalonia and other regions in Spain had a baseline assessment and were randomized to one of the three study arms. We will report the final numbers and the flow diagram of study recruitment in our first paper with the primary outcome results.

### Recruitment and screening

We recruited stroke patients through advertising on social media, publicity, and contacting hospitals and stroke and acquired brain damage patient associations. Before the COVID-19 pandemic, we adopted a local strategy based on publication in the local press and obtaining participants from patient lists of general physicians in the Metropolitan Area of Barcelona. After the pandemic’s start, the recruitment process focused mainly on social networks, expanding the target population to the entire Catalan territory. This change in strategy accelerated the recruitment process and allowed for a larger sample with a more extensive geographic representation.

Interested people contacted the study investigators by telephone, email, or social networks. Patients interested in participating received oral and written detailed information about the study and were asked to fulfill a screening questionnaire to determine if they met the admission criteria. Subsequently, we invited potential participants to a teleconference visit where an evaluator administered the Mini-Mental State Examination (Blesa et al., 2001) and exhaustively reviewed the rest of the study criteria. If candidates were eligible, the information about the study was provided again, and any questions were answered. The informed consent form was signed if the participant agreed to engage in the project. Afterward, an alphanumeric code in the order of arrival was assigned to the participant.

### Assessments

Within two weeks before the interventions started and within two weeks after completing the interventions, we performed medical, cognitive, and physical assessments, neuroimaging, and biological samples collection. When possible, we evaluated patients who dropped out of the study in the follow-up assessment to comply with the intention-to-treat principle.

All the assessments were online except for brain scans, blood tests, and stool sample collection, which were done with extra safety measures at the Hospital Universitari Germans Trias i Pujol (Barcelona, Spain). We conducted online assessments through the Zoom platform (except for medical and physical visits, which were done using a hospital-specific platform). A few days before the assessment, we communicated to the participants the electronic platform which would be used, the link to access the virtual room, and any material they needed to have ready for the visit (e.g., printed documents or objects to do the physical tests).

The assessment timeline is presented in Table 2. Assessors received specific training and a written protocol of the assessment schedule and instructions. The same evaluators carried out the baseline and post-intervention measurements to minimize bias from inter-rater variability.

**TABLE 2:** Assessments schedule.

	Enrollment and screening		Baseline assessments (within 2 weeks prior to the start of the intervention)					Intervention (12 weeks)	Post-intervention assessments (within 2 weeks after the end of the intervention)				
				
	Screening (telematic)	Visit 0 (telematic)	Visit 1 (telematic)	Visit 2 (telematic)	Visit 3 (telematic)	Visit 4 (in-person)	Visit 5 (in-person)		Visit 6 (telematic)	Visit 7 (telematic)	Visit 8 (in-person)	Visit 9 (in-person)	Visit 10 (telematic)
Information of study	✓	✓											
First review of criteria	✓												
Exhaustive review of criteria		✓											
Informed consent form		✓											
Medical story			✓										
General health status			✓							✓			
Neuropsychological tests				✓					✓				
Cognitive scales				✓					✓				
Psychological scales				✓					✓				
Anthropometric measures					✓					✓			
Physical fitness tests					✓					✓			
Lifestyle behaviors scales					✓					✓			
Blood extraction						✓					✓		
Stool sample collection						✓					✓		
Magnetic resonance imaging							✓					✓	
Intervention follow-up diary								✓					
Exercise bracelet								✓					
Qualitative interviews													✓

### Randomization and blinding

After baseline assessments, an independent biostatistician randomized the participants into the three groups through a simple randomizing procedure with an equal allocation ratio (i.e., 1:1:1; 47 individuals per arm) created with a random sequence generator software. We did not use any stratification method due to practical reasons. The randomization sequence was stored on an institutional server and was password protected. Only the person in charge of the randomization, who was not involved in either evaluation or intervention tasks, had access to it.

Group allocation remained blind for all outcome assessors and data analysts, while participants and care providers could not be masked due to the nature of the interventions. Participants received instructions not to discuss any activities related to the intervention with the outcome assessors during the follow-up assessments.

### Interventions

The interventions consisted of five daily sessions per week for 12 weeks and were performed entirely remotely. The mindfulness group received an MBSR program combined with CCT; the PE group received a multicomponent PE program combined with CCT, and the control group only received CCT. The duration of the combined interventions was approximately twice that of the active control group. In the two combined therapy groups, participants did not receive any specific instructions regarding the order in which to perform the interventions.

Mindfulness-based stress reduction and PE interventions included (a) online synchronous sessions guided by expert caregivers and done in groups of 8–12 participants and (b) individual autonomous sessions. In contrast, the control group performed all sessions independently through a computerized telerehabilitation platform. To minimize the confounder effect of benefits from peer interaction and social support in the combined interventions, we invited participants from the same intervention arm to communicate and share experiences through WhatsApp groups.

Before starting the interventions, participants were instructed to use the electronic platforms. Furthermore, they were provided with a manual that collected the main instructions, safety measures to perform the assigned therapies, and a compendium of doubts and frequently asked questions resolved.

#### Mindfulness-based stress reduction program

The intervention followed the scheme and instructions of the official MBSR program designed by Kabat-Zinn (2003). Specifically, within the 12 weeks, there was an introduction session, followed by eight intervention sessions and an intensive practice session interspersed between sessions six and seven. The MBSR intervention took place daily, five days a week. Prototypical weeks had one day with a 150-min online session, while participants performed formal and informal practice for 20–40 min the remaining four days. An accredited mindfulness instructor led the program. Participants were provided with a program dossier that included theoretical explanations and instructions for daily practice and audio recorded by the instructor to guide the meditations. A detailed description of the mindfulness intervention following the template for intervention description and replication (TIDieR) guidelines (Hoffmann et al., 2014) is given in the appendices (see Supplementary Material 1).

#### Multicomponent physical exercise program

The PE intervention was designed following Billinger’s recommendations for stroke survivors (Billinger et al., 2014). It consisted of a 12-week program in which five weekly sessions of 45–60 min were done. Three of these sessions were conducted in groups through video-conferencing technology and led by a physiotherapist and a strength and conditioning specialist. The other two sessions were individualized, and participants were asked to walk. A detailed description of the PE intervention following the TIDieR guidelines (Hoffmann et al., 2014) is given in the appendices (see Supplementary Material 2).

#### Computerized cognitive training program

Computerized cognitive training was carried out using the Guttmann Neuropersonal Trainer (Solana et al., 2015), a computerized telerehabilitation platform. The intervention consisted of a multi-domain task program in which different cognitive processes such as executive function, memory, speed, and attention were included. The platform created personalized training based on the scores obtained in the baseline neuropsychological evaluation, age, and educational level. From the cognitive profile established, it was possible to detect the deficits presented by the participant and thus provide specific rehabilitation exercises. The difficulty of the tasks was adjusted according to the participant’s performance and the therapeutic range (i.e., a percentage of correct answers between 65 and 85%). The training was done individually five days a week, and the duration was 45 min per session.

### Intervention adherence

Intervention adherence was counted as the total number of sessions performed (i.e., the summation of face-to-face and autonomous sessions). Instructors recorded attendance at face-to-face classes, while participants filled in a diary whether they had done the independent sessions and any difficulties or inconveniences they may have experienced. Instructors reviewed the diaries weekly and discussed with participants any incidents or questions that may have arised. In addition, in the case of the PE group, participants were provided with a physical activity bracelet (Fitbit Inspire HR^®^) that they wore the whole intervention.

Furthermore, we controlled whether the participant had already taken any of these interventions before entering the study and any other therapy he/she had followed concomitantly with our interventions. For this reason, we created a questionnaire that participants answered at the end of the trial.

### Safety considerations

Our health professional team reviewed each participant’s baseline assessment before randomization to ensure intervention safety (e.g., all candidates must have a physician’s approval to perform PE before enrollment). If any medical incidence was found in baseline assessments (e.g., in the brain scan or blood test), the participant was informed and redirected to the appropriate health service. In these cases, we decided whether the patient should be excluded from participation based on medical recommendations.

Once assigned to a group and before beginning the interventions, participants received an information session and a written manual with the safety measures they should take to avoid injuries or other potential adverse experiences. They were also instructed to spontaneously report any adverse events throughout the study and to fill in a diary with any difficulties or inconveniences they experienced during or after the sessions. Instructors reviewed the diaries weekly and regularly informed the project manager. Any severe event (e.g., fall, fracture) had to be reported immediately to the Principal Investigator and the Institution’s Research Ethics Committee. Adverse effects will be reported in future manuscripts following official guidelines.

### Tailoring participants’ individual needs

Given the breadth of the eligibility criteria, it was anticipated that the sample would have a wide range of ages and varying degrees of physical and cognitive disability. For this reason, before starting any evaluation or intervention, we determined the individual needs of each participant. Based on these, we decided on the necessary accommodations, which could involve a more comprehensive initial training in digital technologies or specific physical, linguistic, and cognitive adaptations. For example, in the case of people with mild to moderate aphasia who had impaired reading ability, self-administered questionnaires were applied orally by an expert interviewer. Supplementary Materials 1, 2 provide complete information about the adaptations made during interventions.

## Data management and results

### Confidentiality and access to data

Each participant was identified with a dissociated alphanumeric code assigned according to the order of inclusion (i.e., when the patient signed the informed consent). The principal investigator and the project manager kept a confidential record of the participant’s personal information and their assigned identification codes. All trial data was incorporated anonymously in a database stored in an institutional server that requires a password to be accessed (i.e., a strong password consisting of aleatory numbers, upper and lowercase letters, and symbols). Once the database is completed, a double-check process and an assessment of its quality will be carried out. After results publication, data will be shared in anonymized form upon reasonable request.

### Baseline variables

Demographic data (i.e., age, gender, marital status, education, race/ethnicity, employment status, family income) was obtained at baseline through an online questionnaire answered by the participants. Further questionnaires were administered to ascertain cognitive reserve (Rami et al., 2011), personality (Costa and McCrae, 2008), adverse childhood events (World Health Organization, 2020), and expectations about treatment improvements. The Vocabulary and Matrix Reasoning subtests of the Wechsler Adult Intelligence Scale-III (Wechsler, 2001) were administered during the baseline neuropsychological assessment to estimate premorbid intelligence.

The physician in charge of the medical evaluations recorded the existence of current or previous diagnoses apart from stroke, stroke characteristics (e.g., type, evolution time), the specific medication taken by each participant, and smoking and alcohol consumption habits. Moreover, the physician assessed the different neurological symptoms present through the National Institute of Health Stroke Scale (Montaner and Alvarez-Sabín, 2006) and the functionality with the Barthel index (González et al., 2018) and the modified Rankin Scale (Fernández Sanz et al., 2021).

### Outcome variables

#### Primary outcomes

##### Cognitive functioning

All participants completed a comprehensive neuropsychological assessment. We will create z-score composites for each cognitive domain from normalized raw data, as well as a global cognitive function score based on the sum of all domains (see Table 3). Domains will be created following a theoretical-driven approach based on the literature (Lezak et al., 2012). We will consider the changes in cognitive domains and global cognition as primary outcome measures.

**TABLE 3:** Neuropsychological assessment and proposed cognitive composites.

Composite		Test-subtest
Attention–Speed	Attention	Wechsler Adult Intelligence Scale III–Digits Forward (Wechsler, 2001)
		Trail Making Test–A (Tombaugh, 2004)
	Speed	Wechsler Adult Intelligence Scale III–Digit symbol coding (Wechsler, 2001)
Executive functions	Inhibition	Stroop Color and Word Test–Interference (Golden, 2001)
	Flexibility	Trail Making Test–B (Tombaugh, 2004)
	Fluency	PMR phonological verbal fluency (Peña-Casanova et al., 2009)
		Semantic verbal fluency of animals (Peña-Casanova et al., 2009)
	Working memory	Wechsler Adult Intelligence Scale III–Digits Backward (Wechsler, 2001)
Language		Boston Naming Test (15-items) (Goodglass et al., 2001)
Memory	Verbal memory	Rey Auditory Verbal Learning Test–Total learning (Schmidt, 1996)
		Rey Auditory Verbal Learning Test–Delayed recall (Schmidt, 1996)
	Visual memory	Rey-Osterrieth Complex Figure–Memory accuracy (Rey, 2009)
Visuospatial functions		Rey-Osterrieth Complex Figure–Copy accuracy (Rey, 2009)

##### Emotional wellbeing

Depression, anxiety, and stress levels were measured through the Beck Depression Inventory (Sanz et al., 2003) and the Depression Anxiety Stress Scale (Bados et al., 2005). Detailed information on these questionnaires is provided in Table 4. We will consider the changes in these questionnaires’ total scores as primary outcome measures.

**TABLE 4:** Questionnaires.

Name	No. of items	Domain or domains assessed
Beck Depression Inventory-II (Sanz et al., 2003)	21	Depressive symptoms
Childhood Adverse Experience International Questionnaire (World Health Organization, 2020) (baseline only)	36	Adverse childhood experiences
Clinical Outcome in Routine Evaluation-Outcome Measure (Feixas et al., 2012)	34	Psychological distress with four domains: subjective wellbeing, problems/symptoms, general functioning, and risk
Cognitive Reserve Questionnaire (Rami et al., 2011) (baseline only)	8	Cognitive reserve estimation based on proxies
Concomitant Therapies Questionnaire (post-intervention only)	15	Registry of other therapies that the participants follow concomitantly with the study interventions
Demographics and Health History (baseline only)	50	Demographics, medical story, consumption of alcohol and tobacco, COVID-19 data (infection and vaccination), female reproductive health (only in women), data important for microbiome analysis
Depression Anxiety Stress Scale (Bados et al., 2005)	21	Depressive, anxiety, and stress symptoms
Dysexecutive Questionnaire (Pedrero Pérez et al., 2009)	20	Dysexecutive symptoms
Expectancy Questionnaire (baseline only)	10	Expectations about treatment improvements in cognition, emotional wellbeing, physical fitness, and quality of life due to study interventions
Fatigue Assessment Scale (Cano-Climent et al., 2017)	10	Physical and psychological aspects of fatigue
Five Facet Mindfulness Questionnaire (Aguado et al., 2015)	39	Five factors of mindfulness: observing, describing, acting with awareness, non-judging of inner experience, and non-reactivity to inner experience
Food Frequency Questionnaire (Vioque and Quiles, 2003)	93	Frequency consumption of specific foods
Mediterranean Diet Adherence screener (Martínez-González et al., 2012)	14	Adherence to the Mediterranean diet
Memory Failures in Everyday Questionnaire (Montejo Carrasco et al., 2012)	28	Memory failures in daily life
Mindfulness Attention Awareness Scale (Soler et al., 2012)	15	Mindfulness (especially the “acting with awareness” component)
NEO Five-Factor Inventory (Costa and McCrae, 2008) (baseline only)	60	Five factors of personality: openness to experience, conscientiousness, extraversion, agreeableness, and neuroticism
Perceived Changes Questionnaire (post-intervention only)	10	Participants’ subjective experienced changes in cognition, emotional wellbeing, physical fitness, and quality of life due to interventions
Pittsburgh Sleep Quality Index (Royuela Rico and Macías Fernández, 1997)	19	Seven components of sleep quality: subjective sleep quality, sleep latency, sleep duration, habitual sleep efficiency, sleep disturbances, use of sleeping medication, and daytime dysfunction
Profile of Mood States-fatigue subscale (Andrade et al., 2013)	7	Mood states related to physical and psychological aspects of fatigue
Psychological Wellbeing Scales (Díaz et al., 2006)	29	Six factors of psychological wellbeing: self-acceptance, positive relations, autonomy, environmental mastery, purpose in life, and personal growth
Rating Scale for Attentional Behavior (Ponsford and Kinsella, 1991)	14	Attentional behavior in daily life
Reduced Minnesota Leisure-Time Physical Activity Questionnaire (Ruiz Comellas et al., 2012)	6	Physical activity
Stroke-specific Quality of Life Scale (Fernández-Concepción et al., 2008)	38	Eight dimensions of stroke-related quality of life: physical state, communication, cognition, emotions, feelings, basic activities of daily living, ordinary activities of daily living, and family functions
World Health Organization Quality of Life (Abbreviated form) (Lucas-Carrasco, 2012)	26	Four dimensions of quality of life: physical health, psychological health, social relationships, environment

#### Secondary outcomes

##### Psychosocial and lifestyle measurements

We applied a broad range of online questionnaires (see Table 4) to assess changes in subjective cognitive functioning (i.e., dysexecutive symptoms, attention and memory failures in everyday life), additional emotional wellbeing variables, mindfulness, fatigue, functioning, quality of life, and lifestyle behaviors. Furthermore, at the end of the interventions, all participants completed a questionnaire about whether they had experienced changes in cognition, emotional wellbeing, physical fitness, and quality of life, and, if so, they must rate the magnitude of either the improvement or deterioration. In the same questionnaire, we added some questions about the feasibility and acceptability of the interventions.

##### Anthropometric measures and physical fitness

Participants self-reported the values corresponding to height, weight, waist, and hip perimeters. Physical status and functioning (i.e., aerobic capacity, strength, flexibility, and balance) were evaluated with an adaptation of the Senior Fitness Test (Rikli and Jones, 1999). Detailed information on fitness tests is provided in Table 5.

**TABLE 5:** Physical fitness components of the Senior Fitness Test (Rikli and Jones, 1999).

Component	Test
Aerobic endurance	2-min step test
Agility/dynamic balance	8-foot up-and-go test
Flexibility	
Lower body	Chair sit-and-reach test
Upper body	Back scratch test
Strength	
Lower body	30-s chair stand test
Upper body	30-s arm curl test

##### Brain magnetic resonance imaging

All images were obtained with a 3 Tesla scanner (Siemens Magnetom Verio Symo MR B17). Structural, functional, and diffusion sequences were acquired with the parameters described in Table 6. The participants who presented contraindications to performing MRI (e.g., pacemaker, valves, metals in the body, claustrophobia) were not evaluated.

**TABLE 6:** Magnetic resonance imaging (MRI) and biological samples protocols.

MRI protocol	
**Sequence**	**Parameters**

T1-weighted multiplanar reformat sequence	Resolution: 0.9 × 0.9 × 0.9 mm, TR/TE: 1900/2.73 ms, slices: 192
T2-weighted turbo spin-echo sequence	Resolution: 0.7 × 0.5 × 3 mm, TR/TE: 6000/74 ms, slices: 35
T2-weighted turbo inversion recovery magnitude	Resolution: 1 × 0.8 × 3 mm, TR/TE: 9000/99 ms, slices: 44
Susceptible-weighted imaging with T2–fl3d sequence	Resolution: 1.1 × 0.8 × 2 mm, TR/TE: 28/20 ms, slices: 88
Resting state with a gradient echo planar imaging sequence	Resolution: 3.1 × 3.1 × 3 mm, TR/TE: 2000/25 ms, slices: 39, measurements = 240
Diffusion tensor imaging, echo planar imaging	Resolution: 2 × 2 × 2 mm, TR/TE: 10200/89 ms, directions: 64, inverse b0 pulse was added to obtain PA acquisition

**Blood sample protocol**	

**Process**	**Brief explanation**

Collection method	Blood samples were collected in vacutainer tubes containing EDTA disodium (anticoagulant) and were further processed to obtain plasma or frozen at −20°C
Plasma extraction and preparation	Platelet-poor plasma was separated by double centrifugation. Aliquots were frozen at −80°C for following analysis
Plasma analyses	Plasma parameters were measured by ELISA: IL-6, BDNF, IGF-1, Cortisol and VEGF values were obtained using kits from R&D Systems (Minneapolis, MN, USA), and CRP was analyzed by a turbidimetric method using reagents from RAL (Sant Joan Despí, Spain)
DNA extraction	DNA extraction was performed from 3 ml of frozen blood using the Chemagic DNA Blood Kit special [Chemagic MSMI (PerkinElmer, Waltham, MA, USA)]
DNA analyses	Polymorphisms in the APOE gene (single-nucleotide polymorphism rs429358 and rs7412) were determined using TaqMan methodologies

**Stool sample protocol**	

**Process**	**Brief explanation**

Collection method	Fresh stool samples were collected in plastic containers that were immediately put on ice
Preparation and storage	Aliquots were frozen at −80°C for subsequent analysis
DNA extraction	DNA extraction was performed from 200 mg of stool sample in the InviGenius Plus Instrument using the InviMag Universal Kit (Invitek, Berlin, Deutschland)
DNA analyses	The quantitative and qualitative analyses from the DNA was performed by spectrophotometry (Epoch, BioTek, Santa Clara, CA, USA)
SCFA determination	The determination of SCFA was carried out by gas chromatography combined with mass spectrometry

APOE, apolipoprotein E; BDNF, brain-derived neurotrophic factor; CRP, C-reactive protein; DNA, deoxyribonucleic acid; EDTA, ethylenediaminetetraacetic acid; ELISA, enzyme-linked immunosorbent assay; IGF-I, insulin-like growth factor I; IL-6, interleukin-6; MRI, magnetic resonance imaging; SCFA, short-chain fatty acids; VEGF, vascular endothelial growth factor.

##### Blood-sample and stool-sample data

We used a standard blood test to quantify hemograms, biochemical parameters, and lipid profiles. BDNF, IGF-I, vascular endothelial growth factor (VEGF), CRP, IL-6, and cortisol were analyzed in plasma, and genetic biomarkers on APOE were determined in the buffy coat fraction. Stool samples were collected to quantify species and subspecies of the intestinal microbiota, as well as the determination of short-chain fatty acids (SCFA). Specific details of the collection, storage, and analysis of the biological samples are included in Table 6.

### Exit qualitative interviews

We performed a qualitative study on a subset of participants (those with the highest adherence to interventions and willingness to participate) to know their experiences and evaluate the clinical significance of potential changes. We used standard guidelines (O’Brien et al., 2014) to develop the interview script and the data collection and analysis protocol.

### Analysis

#### Power analysis

Sample size calculations were conducted using the Granmo v7.12 software. Accepting an alpha risk of 0.05 and a beta risk of less than 0.20 in a bilateral contrast, 47 participants in each group are required to detect a minimum difference of 0.75 standard deviations between each pair of groups (assuming that there are three) after standardization of the variables to have mean 0 and standard deviation 1. This sample size supports a 20% tracking loss rate. We established a minimum effect size of 0.75 standard deviations by considering the results of meta-analyses studying the effects of mindfulness or PE on cognition or emotional wellbeing in stroke patients (e.g., Abbott et al., 2014; Oberlin et al., 2017; Ali et al., 2021; Sun et al., 2021). The effect sizes reported in these papers range from small-moderate (between *d* = 0.30 and *d* = 0.50) to moderate-large (between *d* = 0.50 and *d* = 1), depending on the intervention and the outcome studied. We used an intermediate effect size that was realistic to the literature and satisfied our trial’s practical and economic possibilities.

#### Statistical analysis

Statistical analysis will be carried out with IBM statistical package for the social sciences (SPSS) Statistics 27 and R environment. The same statistical pipeline will be run twice, in two sequential phases: (1) intention-to-treat analysis considering data from all randomized participants, including those that complete and drop out of the study (in case of missing values, an adequate method of imputation will be executed whenever appropriate, depending on the proportion of missing data and the missingness mechanisms); (2) per-protocol analysis including only those who have had a treatment adherence equal to or higher than 80%. The significant threshold will be set at a two-tailed *p*-value of 0.05, and an appropriate multiple comparisons correction method will be implemented.

Baseline demographic and clinical characteristics of participants in the different intervention groups will be described with central tendency, dispersion, and position measures and compared between the three groups using parametric or non-parametric tests. To address our primary hypotheses, we will use multiple regression models, which will include: (a) the difference between the follow-up and baseline values in the primary outcome measures as the explained variable; (b) the 3-arms intervention as the explaining variable, and (c) other confounding factors as covariates. We will consider sex, age, years of education, time since stroke, and the baseline score as fixed covariates. Moreover, we will contemplate the need to adjust for further unbalanced factors between groups that appear to correlate strongly with the outcomes. In case of violating the linear regression assumptions, transformations of the variables or methods of robust regression will be adopted. We will examine the secondary outcomes with the same pipeline in the per-protocol sample. Although the sample size was not calculated to allow interaction effects, we will examine potential mediator and moderator effects in this sample.

## Discussion

The MindFit Project is a randomized controlled clinical trial that aims to assess the impact of mindfulness and PE combined with CCT on chronic stroke patients’ cognitive and emotional wellbeing. We predict that the three intervention groups will show improvements at the end of the trial, but we expect that these changes will be quantitatively and qualitatively different between groups. We expect that groups that have completed a combination of therapies will show more significant changes than those who have only done CCT. In addition, we hypothesize that there will be a different impact of mindfulness and PE on the cognitive and emotional domains.

Secondly, we plan to study the physiological mechanisms underlying the changes produced by mindfulness and PE through biomarkers to obtain evidence on the biological pathways affected by each intervention and the final effect on the structure and functioning of the brain. A final step in understanding the possible effects of our interventions will be to analyze which personal and clinical variables moderate their impact. In this regard, we will be able to determine which strategies are most suitable for each patient profile and thus lead stroke rehabilitation toward a personalized treatment model.

As a limitation of our study, we highlight the lack of a passive control group that prevents us from determining the efficacy of CCT alone. On the other hand, 3 months of intervention may not be enough to obtain robust and clinically meaningful results. There is a possibility that we will observe changes in biomarkers that are not reflected in behavior. Instead, there may be changes in cognition and emotional wellbeing that are not yet sufficiently established to see their molecular and brain correlates. Despite these limitations, the MindFit Project has the potential and ability to generate new knowledge at multiple levels of evidence, from molecular bases to behavioral changes associated with mindfulness and exercise therapies in chronic stroke patients. In addition, the integration of a qualitative perspective will help us understand the significance of these possible changes and their clinical relevance.

## Dissemination

The results of the MindFit project will be presented in numerous peer-reviewed publications. We will also publish secondary analyses of the clinical trial, and cross-sectional studies will be developed with the participants’ baseline data. The results will also be presented at national and international conferences. Our research group will strive to publish the results regardless of their nature or direction concerning the initial hypotheses.

We will also promote the divulgation of the knowledge obtained: we will organize a closing day for our participants where we will present the main results; we will provide participants with published articles translated into Catalan and Spanish; we will create infographics with the results and publish them through our newsletter and social networks.

## Ethics statement

All participants gave written informed consent following the Declaration of Helsinki. The Bioethics Commission approved the protocol of the University of Barcelona (IRB00003099), the Germans Trias i Pujol University Hospital Research Ethics Committee (PI-17-110), the Healthcare Ethics Committee from the Institut Guttmann, and the Clinical Research Ethics Committee of IDIAP Jordi Gol (19/056-P).

## Author contributions

MM, MD, and AG-M conceptualized the study and contributed to the study design and implementation as principal investigators. AB-G, MA, and RD-A made substantial contributions to the design and content of the trial. NC-V, MB, OC, BF-U, AB, MV, GP, CC, MG, JT, AA, GD, PT-M, JS-R, SD, AP-L, and KE contributed to the design of the trial from their area of expertise. AB-G, MA, DA, NC-V, MB, OC, and SV collaborated to implement specific procedures. AB-G and MA contributed to the protocol’s design, implementation, and writing. All authors contributed to the article and approved the submitted version.

## Abbreviations

APOE: apolipoprotein E
BDNF: brain-derived neurotrophic factor
CCT: computerized cognitive training
CRP: C-reactive protein
DNA: deoxyribonucleic acid
EDTA: ethylenediaminetetraacetic acid
ELISA: enzyme-linked immunosorbent assay
IGF-I: insulin-like growth factor I
IL-6: interleukin-6
MBSR: mindfulness-based stress reduction
MRI: magnetic resonance imaging
PE: physical exercise
SCFA: short-chain fatty acids
SPIRIT: standard protocol items: recommendations for interventional trials
SPSS: statistical package for the social sciences
TIDieR: template for intervention description and replication
VEGF: vascular endothelial growth factor.
