# Supplementary material for: Effects and mechanisms of mindfulness training and physical exercise on cognition, emotional wellbeing, and brain outcomes in chronic stroke patients: Study protocol of the MindFit project randomized controlled trial
Source: Front Aging Neurosci. 2022 Sep 29;14:936077. doi: 10.3389/fnagi.2022.936077 (PMC9557300; doi:10.3389/fnagi.2022.936077)
Supplement: Supplementary file 1 [file Data_Sheet_1.docx]

Supplementary Material 1. Mindfulness Program Description

We followed the Template for Intervention Description and Replication (TIDieR) guide ([Hoffmann et al., 2014](#Hoffmann2014)) in order to describe the mindfulness program in enough detail to allow for its reliable replication.

# Brief name

Online adaptation of the Mindfulness-Based Stress Reduction (MBSR) program for chronic stroke patients.

# Why

## Standardized interventions based on the concept of mindfulness (e.g., MBSR) have grown substantially in recent decades and have been successfully applied in healthy and clinical human populations, with benefits reported in a broad range of variables: mental health, cognition, pain, fatigue, physical health, and quality of life ([Goldberg et al., 2022](#Goldberg2022)). Systematic reviews emphasize the potential utility of mindfulness in treating emotional disturbances ([Lawrence et al., 2013](#Lawrence2013)), fatigue ([Ulrichsen et al., 2016](#Ulrichsen2016)), and sensorimotor functions ([Zou et al., 2018](#Zou2018)) in stroke patients.

# What

## The intervention was based on the MBSR curriculum, initially developed by [John Kabat-Zinn (2003)](#KabatZinn2003) and published in the Authorized Guide edited by [Santorelli et al. (2017)](#Santorelli2017).

## Material

### **Materials used by the instructor to carry out the sessions**

1. A professional Sandberg camera, along with a specific tripod.
2. A Rodes N1100 microphone with an antipop filter and a Focusrite Scarlet 212 sound mixer.
3. PowerPoint presentations with essential elements of theoretical training with graphics and visuals.
4. Document to record attendance.

### **Materials required for participants to follow the sessions**

1. PC with Internet access, camera, and microphone, preferably connected via Ethernet cable. In some cases, Wi-Fi connection and completion of the course using a tablet were allowed.
2. Mat to do the exercises on, and in addition, an armchair, sofa, or mattress, depending on the possibility of mobility of the participant.

### Material delivered to participants to follow the course

1. The course book was organized session by session. Each chapter corresponded to a specific class in the course and included the following sections: (a) introduction to the session; (b) presentation of the theoretical part of the session; (c) practical critical themes of the session; (d) practice guide of the week; (e) additional reading; (f) graphic support material.
2. The audios were provided in MP3 format and were also accessible from the Insight Timer APP. They were delivered in Catalan and Spanish.
3. A weekly record sheet to record the formal and informal practices of the weekdays.
4. A mini-manual with practical topics on how to use the Zoom platform.

## Procedures

The MindFit MBSR program entirely followed the contents of the original curriculum of Jon Kabat-Zin. The only methodological adaptations to the standard MBSR protocol that were made for the MindFit Project are described below.

The standard MBSR has eight consecutive weeks. In the case of the MindFit program, it was lengthened to 12 weeks to match the duration of the physical exercise program (see **Supplementary Material 2**). The following schedule was used (see a synthesized representation in [**Supplementary Table 1.1**](#ST11)):

1. Session 0 was considered one more session in the MindFit program (in the standard MBSR, it is an orientation session with a maximum duration of one hour, which is not included in the eight-week intervention period). The session lasted 90 minutes and followed this structure: (a) introduction and welcome to the course by a representative of the research team; (b) presentation of the detailed content of each session; (c) delivery of course materials; (d) introduction of meditation practice and mindful exercises; (e) training on how to use the Zoom platform; (f) open round of questions and answers.
2. The all-day practice was held a week apart (i.e., the week between classes 6 and 7), increasing by one week the duration of the MindFit program (in the standard MBSR, this session is held on the weekend between weeks 6 and 7). We made this adaptation to avoid overfatigue in our participants (because, in addition to mindfulness, they were doing 45 minutes of cognitive training from Monday to Friday).
3. Two weeks were added in which there was no class (i.e., between classes 3 and 4 and between the all-day practice and class 7). In these weeks without classes, participants were required to continue the formal and informal practice of the previous week. The rationale for introducing these two weeks was to allow participants to consolidate learning and improve and generalize meditation habits in daily life.

| **Supplementary Table 1.1.** Comparison between MBSR standard schedule and our intervention | | | | | | | | | | | | |
| --- | --- | --- | --- | --- | --- | --- | --- | --- | --- | --- | --- | --- |
|  | **Week** | | | | | | | | | | | |
|  | **1** | **2** | **3** | **4** | **5** | **6** | **7** | **8** | **9** | **10** | **11** | **12** |
| **Standard MBSR** | **S1** | **S2** | **S3** | **S4** | **S5** | **S6** | **S7** | **S8** |  |  |  |  |
|  |  |  |  |  |  | **Int. day** |  |  |  |  |  |  |
| **MindFit MBSR** | **S0** | **S1** | **S2** | **S3** | **Rest week** | **S4** | **S5** | **S6** | **Int. day** | **Rest week** | **S7** | **S8** |
| Int. day, Intensive day; S, Session | | | | | | | | | | | | |

# Who provided

The instructor is a mindfulness instructor at the EsMinfulness Institute and Brown University and is part of the MBSR Professional Association of Mindfulness Instructors. He has completed Mindfulness-Based Cognitive Therapy (MBCT) and Cultivating Compassion Training (CCT) programs. He is trained in the neuroscience of meditation and has accompanied his training as a mindfulness and meditation instructor with retreats from different traditions in Europe and Asia. Before the intervention, he had already taught 22 eight-week MBSR courses for stress reduction, dozens of mindfulness programs and workshops, and meditation courses based on mindfulness and neuroscience.

# How

Due to the COVID-19 pandemic, the intervention was online. To adapt the MBSR curriculum to the virtual environment, we followed the Brown University “Teaching MBSR Online” program. Furthermore, the intervention was conducted in groups of between eight and 12 participants. During the classes, people were put into small groups of two or three to discuss specific topics and share their experiences.

# Where

We used the Zoom platform to conduct online classes, as it is the software that best fitted our needs. During the classes, the camera was on throughout the session, except for moments of meditation practice. Participants were instructed to look for a quiet space at home where they felt comfortable. Moreover, we created a broadcast environment with the following physical conditions:

1. A neutral, light-colored background wall, a well-lit environment, and no additional elements on the screen.
2. The VoiceMeeter program was used to treat the sound and noise cancelation gates before sending them to the platform.
3. The meditation practice was accompanied by a soft, neutral sound in the background to help people feel connected to the teacher and to minimize any background noise that might have been there.

In addition to Zoom, discussion forums were encouraged, and we provided participants with spaces to listen, offer support, and share their own experiences and challenges. For example, we created a WhatsApp group with all the people enrolled in the course.

# When and how much

The general schedule of our 12-week intervention is presented in [**Supplementary Table 1.1**](#ST11). The duration of the eight official classes was kept at 2 hours and 30 minutes, as in the standard MBSR protocol. Furthermore, following the guidelines of the original curriculum, the all-day practice lasted 7 hours and 30 minutes, including a) the morning program from 9 a.m. to 1 p.m.; b) lunch from 1 p.m. to 2:30 p.m.; and c) the afternoon program from 2:30 p.m. to 4:30 p.m.

A typical week (with the exceptions already commented on in the section ‘Procedures’) is presented in [**Supplementary Table 1.2**](#ST12). Besides the day of the synchronous class, participants were required to do formal and informal practice during the four resting working days.

| **Supplementary Table 1.2.** Example of a typical week in the MBSR MindFit program | | | | | |
| --- | --- | --- | --- | --- | --- |
|  | **Day** | | | | |
|  | **Monday** | **Tuesday** | **Wednesday** | **Thursday** | **Friday** |
| **Format** | Synchronous session via Zoom | Autonomous sessions (formal and informal meditation practice) | Autonomous sessions (formal and informal meditation practice) | Autonomous sessions (formal and informal meditation practice) | Autonomous sessions (formal and informal meditation practice) |
| **Duration** | 150 minutes | 20-40 minutes | 20-40 minutes | 20-40 minutes | 20-40 minutes |

# Tailoring

## In general

To ensure that participants followed the intervention as well as possible (see also the next section, “[**9. How well**](#_How_well)”), the instructor performed the following actions:

1. Between sessions 0 and 1, he verified that each participant had been able to access practice audios and had all the material available to follow the course.
2. He wrote several emails during each week of the course: (a) the day before the class with the latest materials from the previous session and the instructions for preparation and materials needed for the next session; (b) between 30 and 60 minutes before the class as a reminder; (c) the same day just after the session with the most relevant points of the session; (d) three days after the session with additional material such as readings, stories, or links to videos.
3. He was available 15 minutes before and 15 minutes after the virtual session for those who needed to discuss any topic.
4. He offered the possibility of scheduling a personalized videoconference between sessions.

## Accommodations for cognitive and physical difficulties

Stroke patients often experience some degree of cognitive and physical disability, even in the chronic stages of the disease. For this reason, some accommodations were made to the standard practices of a general MBSR program. However, in our opinion, these changes did not affect the core components of the original intervention designed by Jon Kabat-Zinn.

First, we allowed some participants to be accompanied by a family member during the classes (this is not contemplated in the standard protocol). The caregiver’s role included different functions:

1. To help the participant with technology and the Zoom platform.
2. To transmit and potentiate the messages from the instructor to the participant.
3. To physically support when the participant had to change posture to meditate or perform the Hatha-Yoga movements.
4. To remind the participant to perform both formal and informal daily practice.

For cognitive (e.g., attention or memory) and physical problems (e.g., balance problems, people in wheelchairs), we included some accommodations presented in [**Supplementary Table 1.3**](#ST13).

| Supplementary Table 1.3. Cognitive and physical accommodations | |
| --- | --- |
| For cognitive deficits | **For physical problems** |
| During classes:   - Frequent rest breaks. - Frequent repetition of core concepts and simplified explanations of more complex and conceptual elements of the MBSR curriculum.   After each class:   - Summary hangouts of the session. - Complementary material of the session content (e.g., videos).   Between classes:   - Reminders in the mail from the instructor.   For daily formal practice:   - Shorter versions of the formal practice’s audios. | - Body scan practice was done with the most appropriate posture for each participant (e.g., lying down, sitting, other). - Emphasis on floor-or chair-based mindful movements. - For people in wheelchairs, the upper body practices were done from a sitting position. |

# How well

Adherence to the intervention was assessed as follows:

1. The instructor registered attendance at classes on an Excel sheet. Furthermore, when appropriate, he annotated more qualitative observations that might be valuable to understanding participants’ involvement in the program.
2. The participants had a weekly record sheet to write down the formal and informal practices of the weekdays. This registration sheet had to be delivered during the week following the session.
3. The instructor called the participant between sessions 1 and 2 to assess his/her adherence to the daily practice proposal. Moreover, he made follow-up calls when a participant did not attend the session without notifying his/her absence. When a participant missed a class, the instructor gave him/her a summary explanation of the session and the week’s practice proposal.

# Availability of intervention materials

The corresponding author will share specific intervention material (e.g., meditation audios, record sheets) upon reasonable request.

# References

Goldberg, S. B., Riordan, K. M., Sun, S., and Davidson, R. J. (2022). The Empirical Status of Mindfulness-Based Interventions: A Systematic Review of 44 Meta-Analyses of Randomized Controlled Trials. *Perspect. Psychol. Sci*. 17, 108–130. doi: 10.1177/1745691620968771

Hoffmann, T. C., Glasziou, P. P., Boutron, I., Milne, R., Perera, R., Moher, D., et al. (2014). Better reporting of interventions: template for intervention description and replication (TIDieR) checklist and guide. *BMJ* 348:g1687. doi: 10.1136/bmj.g1687

Kabat-Zinn, J. (2003). Mindfulness-Based Interventions in Context: Past, Present, and Future. *Clin. Psychol. Sci. Pract*. 10, 144–156. doi: 10.1093/clipsy.bpg016

Lawrence, M., Booth, J., Mercer, S., and Crawford, E. (2013). A Systematic Review of the Benefits of Mindfulness-Based Interventions following Transient Ischemic Attack and Stroke. *Int. J. Stroke* 8, 465–474. doi: 10.1111/ijs.12135

Santorelli, S. F., Meleo-Meyer, F., Koerbel, L., and Kabat-Zinn, J. (2017). *Mindfulness-Based Stress Reduction (MBSR) Authorized Curriculum Guide.* Worcester: University of Massachusetts, Medical School Center for Mindfulness in Medicine, Health Care, and Society.

Ulrichsen, K. M., Kaufmann, T., Dørum, E. S., Kolskår, K. K., Richard, G., Alnaes, D., et al. (2016). Clinical Utility of Mindfulness Training in the Treatment of Fatigue After Stroke, Traumatic Brain Injury and Multiple Sclerosis: A Systematic Literature Review and Meta-Analysis. *Front. Psychol*. 7:912. doi: 10.3389/fpsyg.2016.00912

Zou, L., Sasaki, J. E., Zeng, N., Wang, C., and Sun, L. (2018). A systematic review with meta-analysis of mindful exercises on rehabilitative outcomes among poststroke patients. *Arch. Phys. Med. Rehabil*. 99, 2355–2364. doi: 10.1016/j.apmr.2018.04.010
