# Supplementary material for: Effects and mechanisms of mindfulness training and physical exercise on cognition, emotional wellbeing, and brain outcomes in chronic stroke patients: Study protocol of the MindFit project randomized controlled trial
Source: Front Aging Neurosci. 2022 Sep 29;14:936077. doi: 10.3389/fnagi.2022.936077 (PMC9557300; doi:10.3389/fnagi.2022.936077)
Supplement: Supplementary file 2 [file Data_Sheet_2.docx]

Supplementary Material 2. Physical Exercise Program Description

We followed the Template for Intervention Description and Replication (TIDieR) guide ([Hoffmann et al., 2014](#Hoffmann2014)) in order to describe the physical exercise (PE) program in enough detail to allow for its reliable replication.

# Brief name

Online multicomponent PE program for chronic stroke patients.

# Why

# Multiple systematic reviews and meta-analyses have shown the benefits of PE on cognition and emotional well-being across different age groups and health states ([Rebar et al., 2015](#Rebar2015); [Stillman et al., 2020](#Stillman2020)). In stroke, meta-analyses of randomized controlled trials have shown a small to moderate effect of PE on cognition ([Oberlin et al., 2017](#Oberlin2017)), depressive symptoms ([Eng and Reime, 2014](#Eng2014)), and quality of life (Ali et al., 2021).

# What

# The physical exercise intervention was designed following Billinger’s recommendations for stroke survivors ([Billinger et al., 2014](#Bilinger2014)).

## Material

### Materials used by the instructor to carry out the sessions

1. PC with an Internet connection (Ethernet cable or Wi-Fi), camera, and microphone.
2. Document to record attendance at the session.
3. Two chairs (one with arms) to show strength, balance, and agility exercises.
4. Boxes or books to show balance and agility exercises.
5. Water bottles or dumbbells to show strength exercises.

### Materials required for participants to follow the sessions

1. PC with an Internet connection (Ethernet cable or Wi-Fi), camera, and microphone. Completion of the course using a tablet or mobile phone was allowed.
2. Two chairs (one with arms) for strength, balance, and agility exercises.
3. Boxes or books for balance and agility exercises.
4. Water bottles or dumbbells for strength exercises.

### Material delivered to participants to follow the course

1. Document of the Borg scale with an explanation of each score.
2. Document with an explanation of the exercises to be performed in the individual sessions sent weekly.
3. A weekly record sheet to record the activities carried out during individual practice.
4. A physical activity bracelet and a document with the instructions to set up the device and know how it works.
5. A mini-manual with practical topics on how to use the Zoom platform.

## Procedures

The program consisted of a 12-week intervention, with five sessions per week. The intervention targeted the different components of physical fitness: (1) aerobic capacity, (2) strength, (3) agility and balance, and (4) flexibility.

Before starting the program, there was an orientation session with the participants. The objectives and content of this session were as follows:

1. Introduction and welcome to the course by a research team representative.
2. Presentation of the schedule of a typical week in the program.
3. Delivery of course materials.
4. Instructions on:
   1. Material needed to carry out the sessions.
   2. Safety considerations for conducting the sessions.
   3. How to do the strength exercises correctly (grasp the weights accurately without injuring themselves).
   4. How to use the Borg scale to assess the effort made.
   5. How to set up and use the physical activity bracelet.
   6. How to fill out the registration sheet.
   7. Training on how to use the Zoom platform.
5. Open round of questions and answers.

After the orientation session, all weeks had the same structure (see [**Supplementary Table 2.1**](#ST21)). Section “[**7. When and how much**](#_When_and_how)” provides detailed information on the sessions and specific exercises. Within a session, there were always three consecutive blocks:

1. Warm-up, where participants performed joint mobility exercises and activities to raise heart rate and body temperature.
2. The central part was made up of exercises to enhance specific physical fitness components.
3. Cooling down was constituted by stretching and relaxation exercises.

| **Supplementary Table 2.1.** Example of a typical week in the Multicomponent Physical Exercise Program | | | | | |
| --- | --- | --- | --- | --- | --- |
|  | **Day** | | | | |
|  | **Monday** | **Tuesday** | **Wednesday** | **Thursday** | **Friday** |
| **Fitness component** | Strength, agility, and balance | Aerobic capacity | Strength, agility, and balance | Aerobic capacity | Aerobic capacity |
| **Format** | Synchronous session via Zoom | Autonomous sessions (walking) | Synchronous session via Zoom | Autonomous sessions (walking) | Synchronous session (via Zoom) |
| **Duration** | 60 minutes | 45 minutes | 60 minutes | 45 minutes | 45 minutes |

# Who provided

Two instructors led the program. One was a physiotherapist, while the other had a physical activity and sports science degree. Both have experience in rehabilitating patients with acquired brain injury and were under the supervision of a rehabilitation physician.

# How

Due to the COVID-19 pandemic, interventions were available online. As shown in [**Supplementary Table 2.1**](#ST21), three sessions were held synchronously in groups of between eight and 12. The other two sessions were conducted autonomously by the patients.

# Where

We used the Zoom platform to conduct online classes, as it is the software that best fitted our needs. During classes, the camera was mandatory to allow instructors to observe and give feedback on the participants’ performance. In the case of walking sessions, participants walked around their homes.

In addition to Zoom, discussion forums were encouraged, and we provided spaces to listen, offer support, and share their own experience and challenges. For example, we created a WhatsApp group with all the people enrolled in the course.

# When and how much

## Aerobic capacity training

The aerobic capacity was trained in one of the three weekly online classes and during the two autonomous sessions. The videoconference session consisted of 45 minutes of low-impact and cardiovascular-enhanced exercises (see [**Supplementary Table 2.2**](#ST22)). Weeks 1-6 of the program incorporated these exercises at an intensity of 11-12 on the Borg scale and weeks 7-12 at 13-14.

| **Supplementary Table 2.2.** List of aerobic exercises |
| --- |
| Walking in place or moving through space (accompanied by arm movements) |
| Punches forward |
| Punches from the bottom up |
| Alternation of punches forward and walking in place |
| Simultaneous movement of the arms forward and backward |
| Simultaneous movement of arms forward, backward, and sides |
| Simultaneous movement of arms forward, backward, sides, and alternating crosses forward |
| Moving the leg to the side while the other remains stable (alternating legs) and accompanying with forward and upward arm movements |
| Two steps to the right and two steps to the left |
| Raise knee and touch with the opposite hand, alternating legs and arms |
| Alternating movement of the leg to the side while the other remains stable and in front, accompanied by forwarding arms movements |
| Jumping jacks arm movements (only movements of the arms)  Horizontal shoulder adduction-abduction |

In the autonomous sessions, participants were asked to walk around their homes. The volume of walking and its specific intensity (expressed on the 6-20 Borg scale) varied weekly along with the intervention (see [**Supplementary Figure 1**](#SF1)).


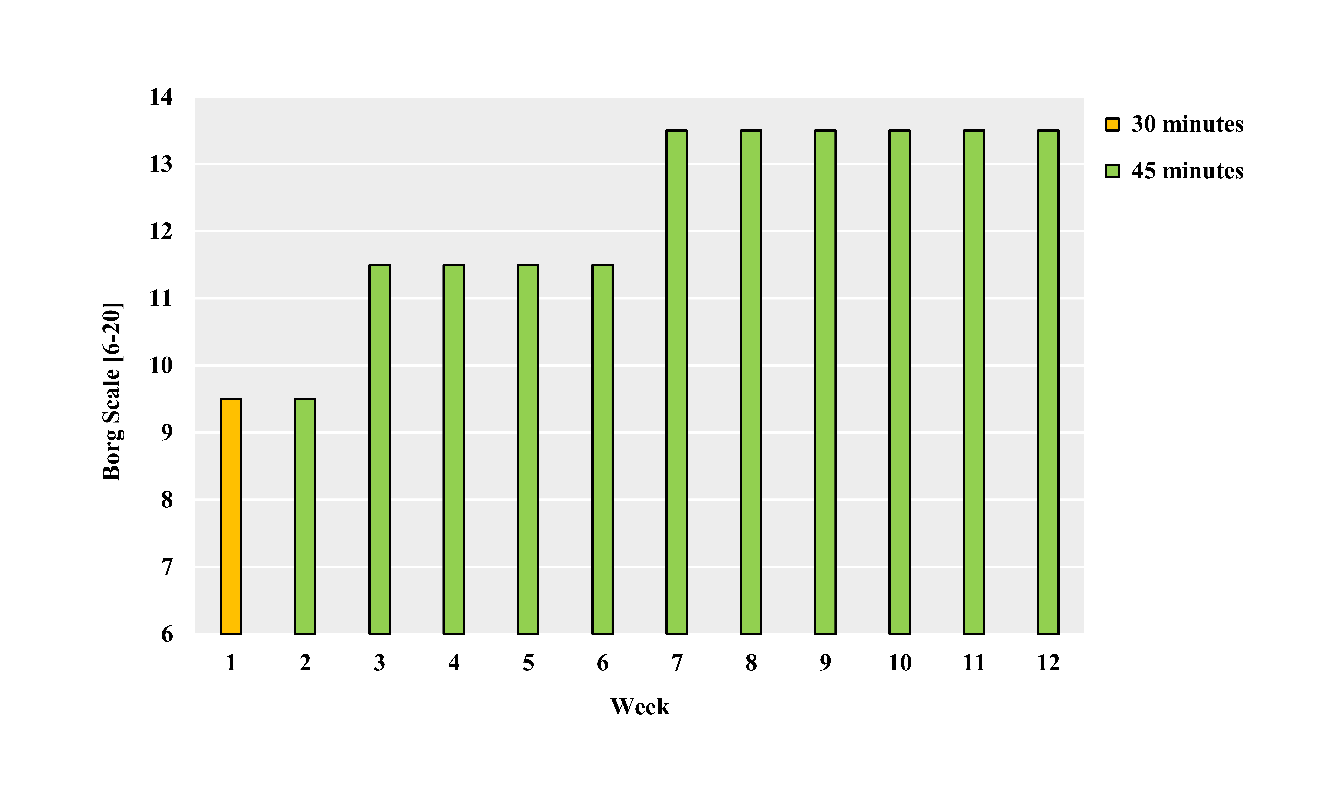
**Supplementary Figure 1**. Intensity of walking along the weeks. The 12-week program started with participants walking for 30 minutes at an intensity of 9-10 on the Borg scale and progressed to walks of 45 minutes with the same intensity in the second week. During the following weeks, the duration of the walk remained unchanged while the intensity increased to 11-12 for weeks 3-6 and 13-14 for the last six weeks.

## Balance, agility, and strength training

The remaining two weekly sessions were conducted using video conference technology and lasted 60 minutes. The objective was to train dynamic balance, agility, and strength.

Within a session, participants performed two balance and agility exercises, followed by six strength exercises (alternating one exercise for the upper limb and one for the lower limb) at moderate intensity (i.e., 12-14 from the Borg scale). These eight exercises were maintained during weeks 1 to 6 of the intervention. Eight new activities were proposed and held during weeks 7-12 of the intervention.

Information on specific exercises and the progression of repetitions and intensity throughout the intervention is presented in [**Supplementary Table 2.3**](#ST23).

| Supplementary Table 2.3. Balance, agility, and strength exercises | | |
| --- | --- | --- |
| First period (weeks 1 to 6) | | |
| Components | **Exercise** | **Repetitions and Intensity** |
| Balance and agility* | **Walk overcoming obstacles** | **Week 1**: 2 sets of 6 obstacles. Go back and forth  **Weeks 2, 3, and 4**: 3 sets of 6 obstacles. Go back and forth  **Weeks 5 and 6**: 3 sets of 7 obstacles. Go back and forth |
|  | **Heel-toe walking** | **Week 1**: 2 sets of 10 steps  **Week 2 and 3**: 2 sets of 15 steps  **Week 4**: 3 sets of 15 steps  **Weeks 5 and 6**: 3 sets of 20 steps |
| Strength | **Squat**  **Chair triceps dips**  **Calf raises.**  **Frontal shoulder elevations**  **Standing Hip Abduction**  **Biceps curl** | **Week 1**: 2 sets of 10 repetitions  **Week 2**: 2 sets of 12 repetitions  **Week 3**: 2 sets of 15 repetitions  **Week 4**: 3 sets of 12 repetitions  **Weeks 5 and 6**: 3 sets of 15 repetitions |
| Second period (weeks 7 to 12) | | |
| Components | **Exercise** | **Repetitions and Intensity** |
| Balance and agility* | **Figure-of- 8 walk** | **Week 7**: 2 sets of 6 repetitions  **Week 8 and 9**: 2 sets of 8 repetitions  **Week 10**: 3 sets of 8 repetitions  **Weeks 11 and 12**: 3 sets of 8 repetitions (increasing walking distance) |
|  | **Stand in tandem** | **Week 7**: 2 sets of 10 seconds  **Weeks 8 and 9**: 2 sets of 15 seconds  **Week 10**: 3 sets of 15 seconds  **Week 11 and 12**: 3 sets of 20 seconds |
| Strength | **Sit-to-Stand**  **Wall Push-Ups**  **Hamstring**  **Lateral shoulder elevations**  **Standing Hip extension**  **Biceps curl** | **Week 7**: 2 sets of 10 repetitions  **Week 8**: 2 sets of 12 repetitions  **Week 9**: 2 sets of 15 repetitions  **Week 10**: 3 sets of 12 repetitions  **Weeks 11 and 12**: 3 sets of 15 repetitions |
| * To increase the intensity of the balance and agility exercises, these can be done without leaning on a support point, or participants can also increase the height of the obstacle in the case of the exercise “Walking overcoming obstacles” | | |

## Flexibility

Flexibility was worked on at the end of each of the five weekly sessions, performing the same upper and lower body stretching exercises throughout the 12 weeks. The exercises were: cross-body shoulder stretch, overhead triceps and shoulder stretch, seated chest stretch, seated hamstring stretch, and calf stretch.

- Weeks 1-3: two sets of 10 seconds for exercise.
- Weeks 4-6: two sets of 15 seconds for exercise.
- Weeks 7-9: two sets of 20 seconds for exercise.
- Weeks 10-12: three sets of 20 seconds for exercise.

# Tailoring

## In general

To ensure that participants followed the intervention as well as possible (see also the next section, “[**9. How well**](#_How_well)”), the instructors performed the following actions:

1. They verified that each participant had all the material to follow the course before the first session.
2. They wrote a reminder email the day before the session with the instructions for preparation and materials needed for the next session.
3. They offered the possibility of scheduling a personalized videoconference between sessions.

## Accommodations for cognitive and physical disabilities

Stroke patients often experience some degree of cognitive and physical disability, even in the chronic stages of the disease. For this reason, some cognitive and physical accommodations were applied.

First, we allowed some participants to be accompanied by a family member during the classes. The caregiver’s role included different functions:

1. To help the participant with technology and the Zoom platform.
2. To transmit and potentiate the messages from the instructor to the participant.
3. To physically support when the participant performed the exercises.
4. To remind the participant to walk in the autonomous sessions.

For cognitive (e.g., attention or memory) and physical problems (e.g., balance problems, people in wheelchairs), we included some accommodations that are presented in [**Supplementary Table 2.4**](#ST24).

| Supplementary Table 2.4. Cognitive and physical accommodations | |
| --- | --- |
| For cognitive deficits | **For physical problems** |
| During classes:   - Frequent rest breaks. - Repetition of the instructions as many times as needed.   Between classes:   - Complementary material of the class and written instructions for autonomous sessions in an easily-understandable manner (e.g., complementing the text description with graphical representations of the strength exercises). - Mail remainders from the instructor. | **Aerobic capacity exercises**:   - People with balance difficulties performed the different cardiovascular-enhanced exercises with one or two hands on the chair. Alternatively, they could sit in a chair and do the exercises from a sitting position if they could not stand the whole session. - People in a wheelchair performed the exercises from a sitting position. For autonomous walking days, we provide them with a 45-minute video with a routine of aerobic exercises to perform from the chair.   **Dynamic balance and agility exercises**:   - If balance was lost, participants had support points in all exercises. - In the walk overcoming obstacles exercise, each barrier’s height was tailored to each participant’s needs.   **Strength exercises**:   - Each strength exercise had three different difficulty levels, so participants could perform the one that best suited their abilities. - Some strength exercises were performed using weights to lift. The weight to be carried was self-determined by the participant under the indication of choosing a weight that could be moved five times more than the number of repetitions requested by the instructor. |

# How well

Adherence to the intervention was registered as follows:

1. The instructors recorded attendance to classes on an Excel sheet. Furthermore, when appropriate, they annotated more qualitative observations that might be valuable to understanding participants’ involvement in the program.
2. Participants had a weekly record sheet to write whether they had done the autonomous sessions and any difficulties or inconveniences they may have experienced. This registration sheet had to be sent to the instructors on Mondays.
3. Participants wore a physical activity bracelet (i.e., a Fitbit Inspire HR® device) throughout the intervention. This device offered daily steps, distance, calories, and continuous heart rate tracking information.

The instructors made follow-up calls when a participant did not attend a session without notifying his absence. When a participant missed a class, the instructors offered a recorded session to the participant to retrieve the session.

1. **Availability of intervention materials**

The corresponding author will share specific intervention material (e.g., detailed information on exercises’ adaptations, record sheets) upon reasonable request.

# References

Ali, A., Tabassum, D., Baig, S. S., Moyle, B., Redgrave, J., Nichols, S., et al. (2021). Effect of exercise interventions on health-related quality of life after stroke and transient ischemic attack: A systematic review and meta-analysis. *Stroke* 52, 2445–2455. doi: 10.1161/STROKEAHA.120.032979

Billinger, S. A., Arena, R., Bernhardt, J., Eng, J. J., Franklin, B. A., Johnson, C. M., et al. (2014). Physical Activity and Exercise Recommendations for Stroke Survivors: A Statement for Healthcare Professionals From the American Heart Association/American Stroke Association. *Stroke* 45, 2532–2553. doi: 10.1161/STR.0000000000000022

Eng, J. J., and Reime, B. (2014). Exercise for depressive symptoms in stroke patients: a systematic review and meta-analysis. *Clin. Rehabil*. 28, 731–739. doi: 10.1177/0269215514523631

Hoffmann, T. C., Glasziou, P. P., Boutron, I., Milne, R., Perera, R., Moher, D., et al. (2014). Better reporting of interventions: template for intervention description and replication (TIDieR) checklist and guide. *BMJ* 348:g1687. doi: 10.1136/bmj.g1687

Oberlin, L. E., Waiwood, A. M., Cumming, T. B., Marsland, A. L., Bernhardt, J., and Erickson, K. I. (2017). Effects of Physical Activity on Poststroke Cognitive Function: A Meta-Analysis of Randomized Controlled Trials. *Stroke* 48, 3093– 3100. doi: 10.1161/STROKEAHA.117.017319

Rebar, A. L., Stanton, R., Geard, D., Short, C., Duncan, M. J., and Vandelanotte, C. (2015). A meta-meta-analysis of the effect of physical activity on depression and anxiety in non-clinical adult populations. *Health Psychol. Rev*. 9, 366–378. doi: 10.1080/17437199.2015.1022901

Stillman, C. M., Esteban-Cornejo, I., Brown, B., Bender, C. M., and Erickson, K. I. (2020). Effects of exercise on brain and cognition across age groups and health states. *Trends Neurosci*. 43, 533–543. doi: 10.1016/j.tins.2020.04.010
